# Interval laparoscopic appendectomy after laparotomy drainage for acute appendicitis with abscess: A case report

**DOI:** 10.1016/j.ijscr.2022.107319

**Published:** 2022-06-18

**Authors:** Toshiyuki Suzuki, Akiyo Matsumoto, Takahiko Akao, Seiji Kobayashi, Hiroshi Matsumoto

**Affiliations:** Department of Surgery, Hanyu General Hospital, Hanyushi Saitama 348-8505, Japan

**Keywords:** CT, computed tomography, CRP, C-reactive protein, POD, postoperative day, WBC, white blood cell, Appendicitis with abscess, Conservative treatment, Drainage, Interval laparoscopic appendectomy, Case report

## Abstract

**Introduction:**

Immediate appendectomy for acute appendicitis with abscess has a high frequency of ileocecal resection and postoperative complications compared with interval appendectomy after conservative treatment. The optimal approach to acute appendicitis with abscess remains controversial.

**Presentation of case:**

A 69-year-old woman was referred to our hospital for abdominal pain. A computed tomography scan revealed an enlarged abscess around the cecum. The diagnosis was perforated appendicitis with abscess, and conservative treatment was performed. Percutaneous drainage was difficult because the abscess was near the intestinal tract. Because of the persistence of symptoms on the fourth day of hospitalization, laparotomy drainage was performed, and the patient's condition improved afterwards. Colonoscopy was performed on an outpatient follow-up to rule out malignant tumors of the colon. Interval laparoscopic appendectomy was performed 3 months after discharge to prevent appendicitis. The postoperative course was uneventful.

**Discussion:**

For this case of acute appendicitis with abscess, conservative treatment such as antibiotic therapy and laparotomy drainage was performed. Laparotomy drainage enabled us to approach the abscess directly and minimized the risk of its spread into the abdominal cavity compared to the laparoscopic approach. Interval laparoscopic appendectomy was more effective and easier for this case of appendectomy, wherein adhesions to the abdominal wall were expected compared to laparotomy.

**Conclusion:**

Conservative treatment approaches, such as drainage and antibiotic therapy, can be first-line for appendicitis with abscesses. Interval laparoscopic appendectomy can be useful to resect the appendix and observe the abdominal cavity.

## Highlights

• For appendicitis with abscess, drainage and antibiotics are the first-line approach.
• Laparotomy drainage is an effective alternative to percutaneous drainage.
• Colonoscopy should be done after conservative treatment to rule out tumors.
• Interval laparoscopic appendectomy is effective for appendicitis with abscess.

## 1 Introduction

The optimal treatment of acute appendicitis with abscess is currently a matter of debate [1]. A meta-analysis by Similis et al. revealed that conservative treatment of acute appendicitis with abscess was associated with significantly fewer overall complications compared to immediate appendectomy [2]. However, another meta-analysis by Furgazzola et al. found that, among children with acute appendicitis with abscess, conservative treatment was associated with lower complication and readmission rates compared with immediate appendectomy [3]. However, the treatment options for appendicitis with abscess in adults remain controversial.

Here, we report an adult case of perforated appendicitis with abscess treated with laparotomy drainage followed by interval laparoscopic appendectomy. This report aims to provide information regarding the treatment for appendicitis with abscess. This case report has been reported in line with the SCARE 2020 criteria [4].

## 2 Presentation of case

**Fig. 1:**
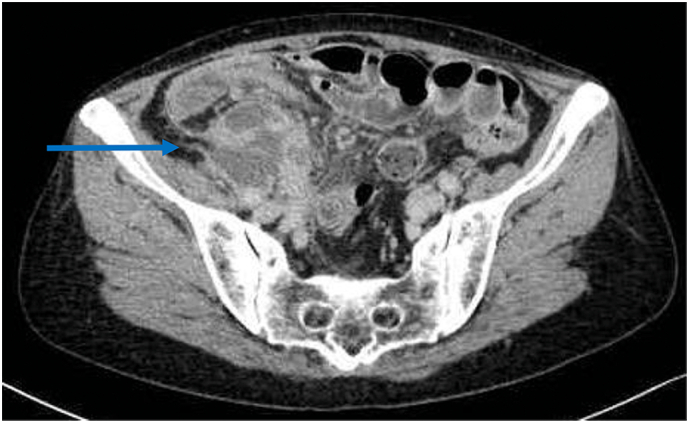
Computed tomography revealed an abscess around the cecum (blue arrow). (For interpretation of the references to colour in this figure legend, the reader is referred to the web version of this article.)

A 69-year-old woman sought consultation at our institution for decreased appetite and lower abdominal pain. The patient was noted to have a fever 10 days ago, which eventually resolved at the time of consultation (Body temperature: 36.1 °C). Lower abdominal pain and tenderness were noted, but there was no rigidity or rebound tenderness. Laboratory tests revealed a white blood cell (WBC) count of 20,790/μl and C-reactive protein (CRP) of 37.6 mg/dL. Computed tomography (CT) scan revealed an abscess around the cecum and ascites in the pelvis (Fig. 1). The presence of appendicitis caused the appendix to collapse and form an abscess. Thus, the patient was diagnosed with perforated appendicitis with abscess. There were no signs of peritoneal irritation, and vital signs were stable. Therefore, conservative treatment with antibiotic therapy was initiated. Despite therapy with meropenem, the patient had a relapse of fever. Abdominal ultrasonography showed an enlarged abscess located behind the intestinal tract, making percutaneous drainage difficult. Therefore, laparotomy drainage was performed by a gastrointestinal surgeon at a district general hospital on the fourth day of hospitalization. The abdomen was opened a via gridiron incision. The area near the cecum was packed with purulent fluid. The purulent fluid was drained, and the peritoneal cavity was thoroughly irrigated with normal saline. A Penrose drain was placed in the abscess cavity. The symptoms improved after the surgery. The drain was removed on the 12th postoperative day (POD), and the patient was discharged on the 15th POD.

Colonoscopy was performed one month after discharge to rule out malignant tumors of the colon. An endoscopic mucosal resection was performed because of a polyp in the cecum. A pathologic examination revealed an inflammatory polyp (i.e., inflammatory granulation tissue), and there were no malignant findings.

**Fig. 2:**
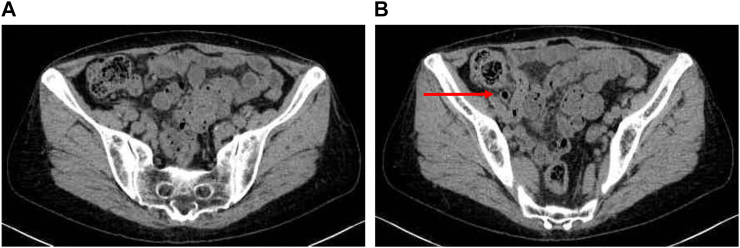
Computed tomography findings. (A) The abscess around the cecum was disappearing. (B) An appendix with gas (red arrow). (For interpretation of the references to colour in this figure legend, the reader is referred to the web version of this article.)

**Fig. 3:**
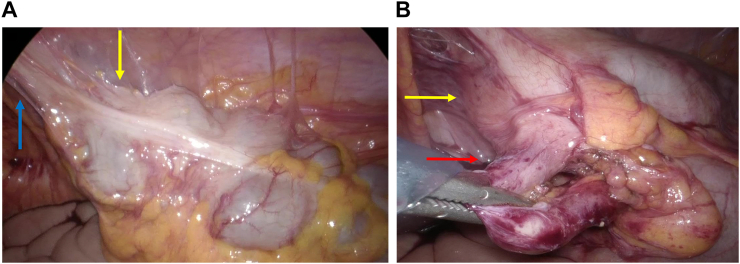
Intraoperative findings. (A) The cecum (yellow arrow) had adhered to the abdominal wall (blue arrow). (B) The adhesion between the appendix (red arrow) and cecum (yellow arrow) was mild. (For interpretation of the references to colour in this figure legend, the reader is referred to the web version of this article.)

On the follow-up, WBC and CRP values were within the normal range. On CT scan detected abscess around the cecum disappearing and appendix with gas (Fig. 2A–B). The risk of occult appendiceal neoplasm increases with increased age, with a 16 % risk in patients aged ≥50 years. Given these findings, interval appendectomy is recommended for all patients with complicated appendicitis and aged ≥30 years. Thus, interval laparoscopic appendectomy was performed by a gastrointestinal surgeon at a district general hospital 3 months post-discharge. The abdominal wall and cecum at the site of the previous incision were adherent (Fig. 3A). The appendix was also mildly adherent to the surrounding tissue, and the appendix stump cutting surgery was easily performed (Fig. 3B). The status of the base of the appendix was apparently normal. The postoperative course was unremarkable, and the patient was discharged on the 2nd POD. The pathological finding was granulomatous appendicitis, and there were no malignant findings.

## 3 Discussion

The proper management of perforated appendicitis with abscess is a clinically important issue [1]. It is technically demanding to perform immediate surgery for patients with acute appendicitis with abscess due to the distorted anatomy and difficulty in closing the appendix stump, with occasional ileocecal resection [5]. Moreover, it is also associated with a higher morbidity compared to nonsurgical treatment (i.e., drainage and/or antibiotic therapy) [5]. Some reports have noted the efficacy of appendectomy for appendicitis with abscess [6], [7], [8]. However, because it was difficult to close the appendix stump, antibiotic therapy was done instead, in order to avoid ileocecal resection and to reduce postoperative complications.

In some cases of appendicitis with abscess, antibiotic therapy alone is not sufficient and case drainage may be effective [9]. Abscess drainage can be done through percutaneous, laparotomy, and laparoscopic methods. In the present case, percutaneous or CT-guided drainage was difficult due to the possibility of intestinal injury at the abscess puncture site during the procedure on abdominal ultrasonography. Laparotomy drainage was performed in this case because it was an easier method. It provides direct access with the abscess cavity compared to laparoscopic drainage, and therefore dose not spread the abscess into the abdominal cavity. No complications occurred after laparotomy drainage.

Interval appendectomy after successful conservative treatment for appendicitis with abscess remains controversial. The rate of recurrence after conservative treatment for perforated acute appendicitis and phlegmon ranges from 12 % to 24 % [10], [11]. To avoid this high chance of recurrence, some recommend regular selective interval appendectomy after initial conservative management [1]. Therefore, interval laparoscopic appendectomy was performed in this case.

Laparoscopic surgery was chosen in order to avoid adhesions at the incision site of the first laparotomy drainage. In fact, the abdominal wall and cecum were highly adherent at the site of the previous incision (Fig. 3A). It is believed that if the laparotomy approach was chosen, it could have been difficult in this situation. The cecum might have been damaged, and it may have been difficult to find the appendix. Conversely, the laparoscopic approach was particularly effective in this case wherein the cecum and abdominal wall were adhered to. It was also easier to search for the appendix.

Low postoperative complications are one of the reasons for choosing interval surgery over immediate surgery. Immediate surgery in acute appendicitis with abscess is prone to postoperative ileus and residual abscess due to the presence of the abscess. Fortunately, no complications occurred after interval laparoscopic appendectomy in this case. Treatment of the abscess via antibiotic therapy or drainage followed by surgery may prevent complications related to the abscess and extended resection, such as ileocecal resection.

Interval surgery was also chosen because it can be performed alongside a colonoscopy while waiting. Renteria et al. reported that the rate of unexpected malignancy was 3 % in of elderly (mean age: 66 years) and 1.5 % of young (mean age: 39 years) patients who underwent appendectomy as primary treatment for acute appendicitis [12]. Jonge et al. reported that adult patients undergoing interval appendectomy can be diagnosed with an appendix neoplasm in up to 11 % of cases, in contrast to only 1.5 % of patients undergoing immediate appendectomy [13]. Recently, a randomized control trial by Mällinen et al. comparing interval appendectomy and follow-up with magnetic resonance imaging after initial successful conservative treatment of periappendicular abscess was prematurely terminated because of ethical concerns. During their interim analysis, there was an unexpected finding of a high rate of neoplasms (17 %), with all neoplasms in patients older than 40 years [14]. Therefore, tumors can actually be one of the causes of appendicitis with abscess. This means that for patients with appendicitis with abscess aged >40 years, conservative treatment should be done alongside colonoscopy to rule out malignant findings. The excision range should then be decided based on the presence or absence of malignant findings.

In this case, laparotomy drainage and interval laparoscopic appendectomy were performed, suggesting a risk of postoperative complications associated with two surgical procedures. However, no postoperative complications were observed. The proper choice of surgical approach can minimize postoperative complications. Thus, even two surgical procedures can be acceptable even for old age patients, such as this case.

## 4 Conclusion

Immediate appendectomy for acute appendicitis with abscess is associated with higher morbidity. Thus, conservative treatment by laparotomy drainage and antibiotic therapy should be performed in such cases. Subsequent colonoscopy should also be performed because of the possibility of malignant findings in patients aged >40 years. Interval laparoscopic appendectomy is effective to easily resect the appendix and observe the abdominal cavity.

## Consent

Patient consent form was signed by the patient. Written informed consent was obtained from the patient for publication of this case report and accompanying images. A copy of the written consent is available for review by the Editor-in-Chief of this journal on request.

## Declaration of competing interest

All authors do not have any conflicts of interest.
